# Long-Term Stability of Glycopyrrolate Oral Solution Galenic Compound at Different Storage Conditions

**DOI:** 10.3390/pharmaceutics16081018

**Published:** 2024-07-31

**Authors:** Barbara Bellich, Martina Franzin, Debora Curci, Mario Cirino, Alessandra Maestro, Giada Bennati, Gabriele Stocco, Gianpiero Adami, Natalia Maximova, Domenico Leonardo Grasso, Egidio Barbi, Davide Zanon

**Affiliations:** 1 Institute for Maternal and Child Health, IRCCS “Burlo Garofolo”, Via dell’Istria 65/1, 34137 Trieste, Italy; barbara.bellich@burlo.trieste.it (B.B.); martina.franzin@burlo.trieste.it (M.F.); debora.curci@burlo.trieste.it (D.C.); alessandra.maestro@burlo.trieste.it (A.M.); giada.bennati@burlo.trieste.it (G.B.); gabriele.stocco@burlo.trieste.it (G.S.); natalia.maximova@burlo.trieste.it (N.M.); domenicoleonardo.grasso@burlo.trieste.it (D.L.G.); egidio.barbi@burlo.trieste.it (E.B.); davide.zanon@burlo.trieste.it (D.Z.); 2 Department of Medical, Surgical and Health Sciences, University of Trieste, 34149 Trieste, Italy; 3 Department of Chemical and Pharmaceutical Sciences, University of Trieste, Via L. Giorgieri 1, 34127 Trieste, Italy; gadami@units.it

**Keywords:** pharmaceutical, drug compounding, drug stability, pharmacopoeia, safety, glycopyrrolate, clinical galenic, pediatrics, sialorrhea

## Abstract

Glycopyrrolate is a competitive muscarinic receptor antagonist used in the treatment of sialorrhea, especially in pediatrics. Degradation research was conducted to better understand the stability of the active pharmaceutical ingredient (API). Using an HPLC-UV method, we evaluated the chemical stability of the oral solution of the galenic compound glycopyrrolate 0.5 mg/mL under different storage conditions. Method validation was performed according to the International Council for Harmonization (ICH) Q2(R2) guidelines. The results of the stability study of the galenic compound in different storage conditions, with the exception of those stored in glass containers at 45 °C for more than 3 months, were stable (100 ± 10% of the nominal concentration). The aim of this work was to study the stability of the galenic compound glycopyrrolate in two different types of containers and at three different storage temperatures. Glycopyrrolate showed degradation beyond the limits only in glass at 45 °C and after 2 months of storage. The results indicate that oral liquid dosage forms of glycopyrrolate are stable for at least 210 days when stored at room temperature or at 4 °C, in glass or PET, for at least 7 months, maintaining product quality according to the standards established by the European Pharmacopoeia, ensuring long-term coverage for pediatric patient therapies.

## 1. Introduction

**Figure 1:**
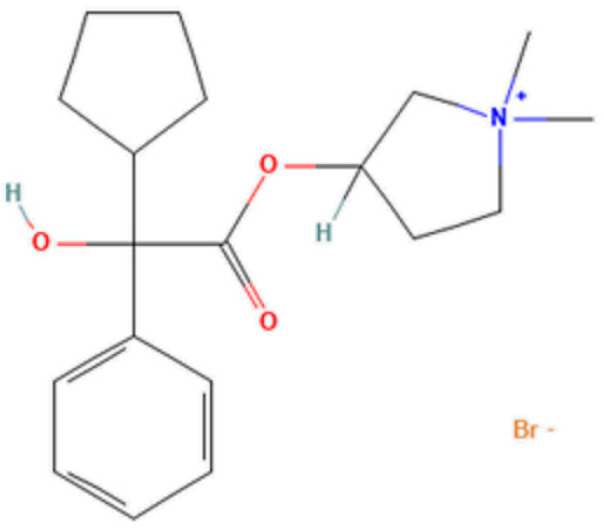
Glycopyrronium bromide IUPAC: (1,1-dimethylpyrrolidin-1-ium-3-yl) 2-cyclopentyl-2-hydroxy-2-phenylacetate;bromide (https://pubchem.ncbi.nlm.nih.gov/compound/11693 accessed on 19 July 2024).

Glycopyrrolate (3-[(2-cyclopentyl-2-hydroxy-2-phenylacetyl)oxy]-1,1-dimethylpyrrolidin-1-ium and bromide as counterion (Figure 1)) is a synthetic anticholinergic agent with a quaternary ammonium structure. It is a competitive antagonist of the M1–M5 muscarinic acetylcholine receptors [1]. It therefore reduces excessive pharyngeal, tracheal, and bronchial secretions. It is used in the treatment of sialorrhea by blocking the muscarinic acetylcholine receptors, M1 and M3, in the salivary glands [2,3,4]. Approximately 35% of pediatric patients suffer from problems related to sialorrhea (caused by conditions of cerebral palsy, muscular/neuromuscular disorders, etc.); sialorrhea is a social problem that has a strong impact on the social life of patients [5]. Currently, in Italy, there is only one approved drug on the market, containing 400 micrograms/mL of glycopyrronium bromide equivalent to 320 micrograms of glycopyrronium in oral solution, indicated for the symptomatic treatment of severe sialorrhea (chronic hypersalivation pathology) in children and adolescents of the same age or more than 3 years with chronic neurological diseases. The shelf life of this approved pharmaceutical product is 2 months after first opening. Degradation studies were performed to gain a better understanding of the stability of the active pharmaceutical ingredient (API) and to recommend data on degradation pathways and degradation products. No research data were found in the literature for the estimation of glycopyrrolate by HPLC in oral solution form. The objective of this work was to develop a stability-indicating HPLC-UV for the analysis of glycopyrrolate in oral compound. The compound was exposed to forced degradation under thermal conditions as described. The concentration selected for the stability study was 0.5 mg/mL; this concentration was used for the compound formulation. The aim of this study was to evaluate the stability of the formulation over time to guarantee a quality galenic product, even near the end of the validity period according to the standards set by the European Pharmacopoeia, guaranteeing wider coverage for chronic therapies of pediatric patients and better organization for production in the galenic laboratory.

## 2. Materials and Methods

### 2.1. Chemicals and Reagents

**Figure 2:**
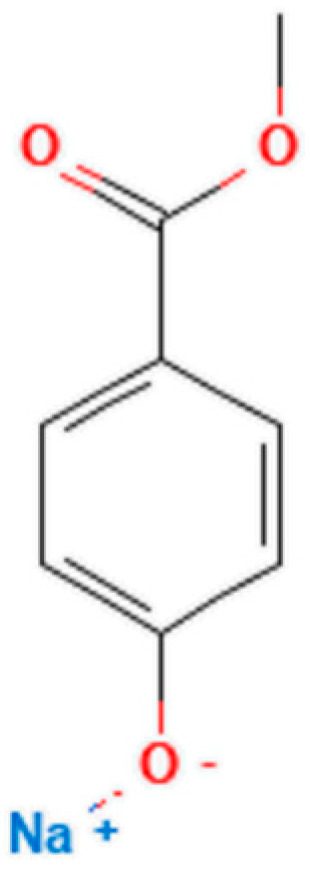
Nipagin sodium, also methylparaben sodium, IUPAC: sodium;4-methoxycarbonylphenolate (https://pubchem.ncbi.nlm.nih.gov/compound/23663626 accessed on 19 July 2024).

All chemicals and reagents used were of analytical grade. Glycopyrrolate was purchased from Fagron Italia S.r.l. (Bologna, Italy). Nipagin sodium salt (Figure 2), sodium phosphate monobasic monohydrate, sodium phosphate dibasic anhydrous, and granulated sucrose for the galenic compound were purchased from Farmalabor S.r.L. (Milan, Italy). Pure water was obtained from a Milli-Q system (Millipore, Darmstadt, Germany). Acetonitrile, potassium phosphate monobasic, and azathioprine were purchased from Merck (Milan, Italy). The filter paper (particle retention: 10–20 µm) was purchased from VWR (Milan, Italy).

### 2.2. Galenic Compound

The galenic glycopyrrolate compound was produced by the Pediatric Institute Pharmacy, as previously described [6]. Briefly, the galenic compound was compounded using glycopyrronium bromide solution with a final concentration of active substance of 0.5 mg/mL, as well as the preservative nipagin (0.05% *w*/*v*), which isan alkyl ester of 4-hydroxybenzoic acid called methylparaben, well known to be used as preservatives in pharmaceuticals, cosmetics, and food products, owing to its antifungal effect, providing a broad spectrum of coverage for effective microbiological stability, as well as the sweetening agent sucrose (10% *w*/*v*) and a phosphate buffer to obtain a syrup with a pH range around 5.6. The formulation was compounded in 100 mL amber glass and amber PET plastic. The low drug concentration permitted the syrup to remain sweet for oral administration [7,8].

### 2.3. Working Solutions

All working solutions were prepared fresh the day of the analysis. In particular, glycopyrrolate solution (0.5 mg/mL) and the internal standard (IS) azathioprine (0.1 mg/mL) were prepared in MilliQ water. Also, a solution of nipagin (0.03 mg/mL) was prepared in MilliQ water.

### 2.4. Method Development

A High-Performance Liquid Chromatography-Ultraviolet (HPLC-UV) method for the quantification of glycopyrrolate was developed using the instrument Agilent 1260 Infinity I equipped with a quaternary pump, UV spectrometer, and a thermostated (10 °C) autosampler (Agilent Technologies, Milan, Italy). The column adopted was a Zorbax Eclipse Plus C18 4.6 × 150 mm with a particle size of 5 µm (Agilent Technologies, Milan, Italy). Chromatographic separation was obtained after elution of mobile phase A (30 mM potassium phosphate monobasic) and B (acetonitrile) in gradient mode. In detail, a flow rate of 1.5 mL/min was maintained for all the chromatographic runs (12 min), and samples were eluted using the following program: 0–6 min isocratic 80% A and 20% B, 6.0–9.0 min linear gradient from 20% B to 60% B, 9.0–10.0 linear gradient from 60% to 20% B, and 10.0–12.0 min isocratic 80% A and 20% B. The column oven was set at 20 °C, and the column temperature was 25 °C (room temperature). The injection volume was 25 µL. The wavelength set was 210 nm. Sample preparation consisted in dilution 1:20 in MilliQ water (25 µg/mL) and addition of 10 µL of IS before the injection in the instrument. Calibrators (CAL), as well as quality controls (QC), were prepared through dilution of pure standard of glycopyrrolate. Different working solutions were used for the construction of calibration curves and for the preparation of QC.

### 2.5. Method Validation

Method validation was carried out according to the International Council for Harmonization (ICH) guidelines Q2(R2) on validation of analytical procedures [9].

Selectivity and specificity of the analytical method were determined by injection of pure standards (i.e., glycopyrrolate, nipagin, and the IS azathioprine) to check for possible interference due to overlapping signals.

Linearity of the instrumental response of the analyte glycopyrrolate was assessed through the construction of at least 3 calibration curves in 3 analytical sessions. In detail, taking into account the sample dilution, the working range tested was 10–80 µg/mL (*n* = 6).

Sensitivity was evaluated based on determination of the lower limit of detection (LOD) and quantification (LOQ). In particular, the validation of these lower range limits was performed through dilution of the lowest point of calibration. The lower detectable concentration of analyte was defined as the LOD, whereas the lower concentration that can be accurately determined was defined as the LOQ.

Accuracy and precision, expressed as percentage of accuracy (ACC%) and coefficient of variation (CV%), were assessed testing 3 levels of QC intra-daily (3 times) and inter-daily (in 3 different runs). The three levels of concentration were 12.5 µg/mL (low QC (LQC)), 22.5 µg/mL (medium QC (MQC)), and 35 µg/mL (high QC (HQC)).

### 2.6. Stability Study by HPLC-UV

Stability of glycopyrrolate solutions was evaluated by the HPLC-UV method developed and validated as reported above.

The galenic compound was dispensed into three amber glass containers and three amber polyethylene terephthalate (PET) containers. Each type of container was stored at three different temperatures (4, 25, and 45 °C), and the stability was evaluated up to 7 months. In particular, at determined time intervals (24–48–72 h, 1–2 weeks, from 1 to 7 months), the sample was analyzed in triplicate. Glycopyrrolate was considered stable, when the concentration varied less than the ±10% of the nominal value according to the directives set out in the European Pharmacopoeia [10].

### 2.7. Identification of Degradation Products by Liquid Chromatography Coupled with Mass Spectrometry (LC-MS) Analysis

Dilutions of galenic compound stored in a glass container and kept at +4 °C and +45 °C were analyzed using the instrument Ultra-High-Pressure Liquid Chromatography Vanquish Neo UHPLC system (Thermo Fisher Scientific, Milan, Italy) coupled to mass spectrometry Exploris 240 (Thermo Fisher Scientific, Milan, Italy). Briefly, the components of the compound were separated by eluting on an Acclaim PepMap RSLC C18 column (150 mm × 1 mm, particle size 2 μm Thermo Fisher Scientific) using an aqueous phase (A) of 0.1% formic acid and an organic phase (B) of acetonitrile 0.1% formic acid. The flow rate was set at 80 µL/min, starting with 1% B until 100% B. The total chromatographic run lasted 13.5 min. The column oven was set at 35 °C, the autosampler was set at 7 °C, and the injection volume was 3 μL. The data acquisition was carried out with the software Thermo Scientific Xcalibur 4.7.

The ESI source operated in positive mode with the following parameters: ion transfer tube temperature: 300 °C, spray voltage (positive ion): 3200 V, sheath gas: 35, and aux gas: 10 (arbitrary units).

Acquisitions were performed in positive ion polarity mode. Acquisition in full scan mode was as follows: scan range 100–1000 *m*/*z*, 60,000 resolution at *m*/*z* 200, normalized AGC target 100%, auto maximum injection time, and intensity threshold 5 × 10^5^. Data-dependent MS2 was as follows: auto *m*/*z* range, stepped HCD normalized collision energy (20, 40, 60), 45,000 resolutions at *m*/*z* 200, normalized AGC target 100%, and auto maximum injection time isolation window *m*/*z* 2. Internal calibration was performed prior to each analysis sequence. Five replicates of each sample were used for statistical analysis.

### 2.8. Statistical Analysis

The HPLC-UV chromatograms were recorded and analyzed with the software Agilent OpenLab Software version 2.0 (Agilent Technologies, Milan, Italy). Calibration curves were fit by linear regression without weighting and forcing the line through the origin. Glycopyrrolate quantification was obtained by normalization of the response of the analyte on the one of the IS and by interpolation with the calibration curve. The mean concentration value, as well as the standard error (SE) (ratio between the standard deviation and the square root of the number of replicates), related to the glycopyrrolate stability study was calculated on three independent triplicates.

The LC-MS data were processed and analyzed using the Compound Discoverer software version 3.3 (Thermo Fischer Scientific, Milan, Italy). The rigor of the metabolite identification was classified according to the Metabolomics Standards Initiative (MSI) [11].

The statistical analysis was carried out on the ratio between the mean area of glycopyrrolate, or its derived compound, in the compounds stored at 45 °C and 4 °C, taking the latter as the control. The *p*-value was calculated by a two-tailed Student’s *t*-test.

## 3. Results

### 3.1. Method Validation

#### 3.1.1. Specificity

**Figure 3:**
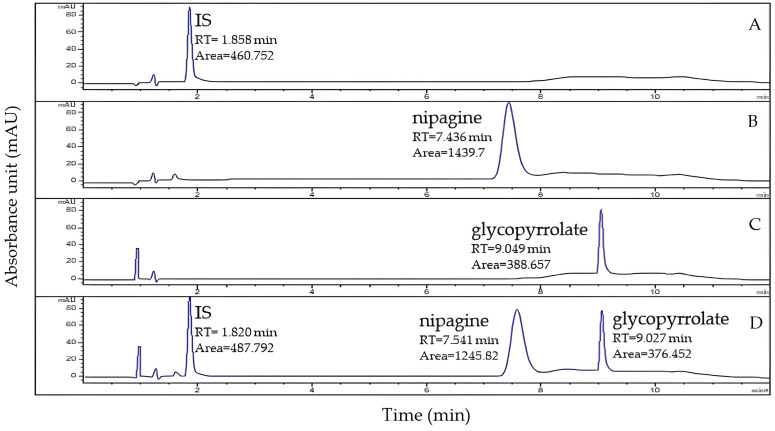
Chromatograms related to the HPLC-UV analysis of the IS azathioprine (**A**), the preservative nipagin (**B**), the glycopyrrolate (**C**), and the galenic compound added with IS (**D**).

As the galenic compound contains the active substance glycopyrrolate and the preservative nipagin, the proposed method was developed and was used to ascertain the potential interference of nipagin in the chromatographic analysis. Figure 3 represents the chromatograms corresponding to the IS azathioprine, the preservative nipagin, and the analyte glycopyrrolate. The retention times were 1.8, 7.9, and 9.1 min, respectively. Of note, no interference at the retention time of glycopyrrolate was observed, thus confirming the specificity of the method for the quantification of glycopyrrolate in this galenic compound.

#### 3.1.2. Linearity

Application of the linear regression model on the calibration curves analyzed in three analytical sessions succeeded in confirming linearity in the range of the concentrations tested (10–80 µg/mL). Of note, the coefficient of determination (R2) for all the three calibration curves was greater than 0.99. Moreover, the percentages of accuracy (ACC) calculated on the nominal concentration of calibrators (CAL) resulted in accurate values (100 ± 15% of the nominal concentration) (Table 1).

**Figure 4:**
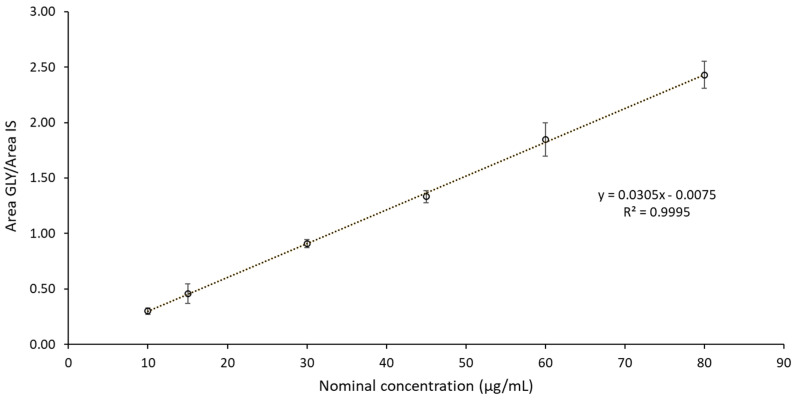
Linearity assessment with error bars and fitting results—mean values from 3 replicates.

The linearity assessment with error bars and fitting results can be seen in Figure 4.

#### 3.1.3. Sensitivity

The proposed analytical method evaluated the lower limit of quantification (LOQ) and of detection (LOD) as measurements of sensitivity. In detail, diluting twice the lowest calibration point resulted in an accurate value (89.38% of the nominal value), thus determining the concentration of 5 µg/mL as the LOQ. Instead, 1 µg/mL corresponded to the LOD.

#### 3.1.4. Accuracy and Precision

To estimate the accuracy and the precision, in terms of repeatability, three quality controls (QC) with different concentrations were evaluated intra-daily (in triplicate) and inter-daily (in three analytical sessions). Table 2 reports ACC, as well as the percentages of coefficient of variation (CV), to express precision. It is noteworthy that the values related to the accuracy of the measurement were in the accepted range (100 ± 15% of the nominal value). Additionally, the intra-day and inter-day CV (%) did not exceed the threshold value of 15%.

### 3.2. Stability Assessment

**Figure 5:**
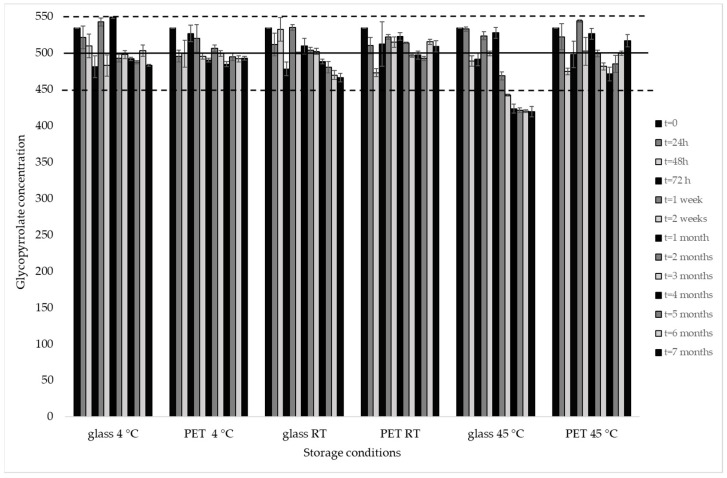
Mean concentration (μg/mL) of glycopyrrolate in galenic compound dispensed in glass or PET containers and stored at +4 °C, room temperature (RT), and +45 °C. Full line represents the initial nominal concentration; dashed line is the lower limit of acceptable concentration according to the directives set out in the European Pharmacopoeia.

The results of the stability study, including the mean concentration values, the standard error, and the ACC, are reported in Table 3. All measurements of galenic compound in different storage conditions, except those stored in glass containers at 45 °C longer than 3 months, resulted in being stable (100 ± 10% of the nominal concentration) (Figure 5).

### 3.3. LC-MS Analysis of Degraded Compound

The results obtained from LC-MS (Table 4) evidenced the presence of glycopyrrolate, the derived compound metabolite M9, and α-cyclopentil mandelic acid (C_13_H_16_O_3_), classified, respectively, as 1 (identified compound) and 2 (putatively annotated compound based upon spectral similarity with online libraries) indexes according to MSI. As shown in Table 5, the active compound glycopyrrolate resulted in a decrease (around −25%), whereas the putatively identified degraded compound increased (around +20%) in the galenic compound stored at 45 °C, in comparison with the one stored at 4 °C. The kinetics of the decomposition is a first-order reaction.

**Figure 6:**
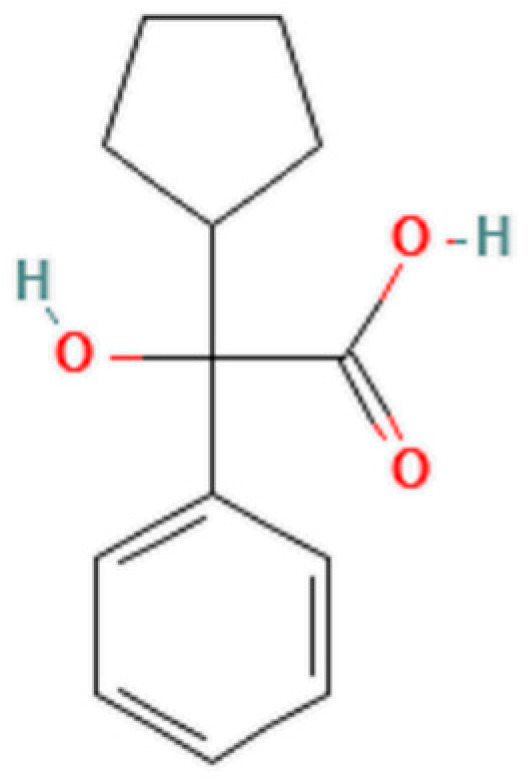
Alpha-cyclopentylmandelic acid, IUPAC: 2-cyclopentyl-2-hydroxy-2-phenylacetic acid (https://pubchem.ncbi.nlm.nih.gov/compound/98283 accessed on 19 July 2024).

A glycopyrrolate solution was investigated by LC-MS analysis; the molecule was identified as ion [M]^+^ with an *m*/*z* value of 318.2062; the fragmentation pattern evidenced the presence of the ion with *m*/*z* = 116.1069, which is diagnostic for glycopyrrolate identification [12]. According to Santus et al., 2017 [13], glycopyrrolate undergoes a non-enzymatic hydrolysis, whose product is the metabolite M9. This compound was identified by comparison of the MS2 from the precursor ion and it corresponds to a-cyclopentylmandelic acid. Similarly, Chawla et al., 2021 [14], reported that glycopyrrolate degrades in a compound named as compound C (the same as metabolite M9 in other study [13]) and a 1,1-dimethyl-3-hydroxy-pyrrolidinium bromide, as shown in Figure 6. This compound (C_6_H_14_NO) with an *m*/*z* value of 116.1070 was also detected in our samples by LC-MS. Therefore, it is reasonable that the hydrolysis of glycopyrrolate is the main degradation process occurring.

## 4. Discussion

The aim of this work was to study the stability of glycopyrrolate galenic compound in two different types of containers and at three different storage temperatures. This is an important choice to guarantee better compliance for families and children. Glycopyrrolate showed degradation beyond the limits (according to the directives established by the European Pharmacopoeia) only in glass at 45 °C and after 2 months of storage (it should be underlined that this is an extremely high temperature). The proposed HPLC-UV method efficiently separated and showed all the analyzed peaks confirming the system suitability. The coefficient of determination, R2, was greater than 0.99. The results obtained from LC-MS highlighted the presence of the metabolite of the derived compound M9 and α-cyclopentyl mandelic acid (C_13_H_16_O_3_) (compound spectral similarity with online libraries) according to MSI. The strength of this method is confirmed by the results, despite the presence of excipients. A second hypothesis on potential degradation is based on the effect of the drug’s concentration. No aggregation nor precipitate was observed. The results indicate that oral liquid dosage forms of glycopyrrolate (0.5 mg/mL) were stable for at least 210 days when stored at room temperature, in either glass or PET. The physical appearance of the dosage forms did not change.

## 5. Conclusions

According to the stability study conducted, it was seen that the degradation of glycopyrrolate oral solution showed long-lasting stability, even at room temperature. Any oral compound can be stored both at 4 °C and at room temperature for at least 7 months in both glass and PET containers while maintaining the quality of the product according to the standards set by the European Pharmacopoeia, ensuring long-term coverage for pediatric patient therapies. The validation was completed, confirming the ICH Q2 (R2) recommendations, and the process proved to be selective, specific, precise, accurate, and robust, providing linear responses in concentration gradation.

## Figures and Tables

**Table 1:** Calculated concentrations, as well as calculated intra-day and inter-day percentages of accuracy (ACC), related to calibrators (CAL).

Standard	Nominal Concentration (µg/mL)	First Analytical Session		Second Analytical Session		Third Analytical Session		Inter-Day ACC (%)
		Calculated Concentration (µg/mL)	Intra-Day ACC (%)	Calculated Concentration (µg/mL)	Intra-Day ACC (%)	Calculated Concentration (µg/mL)	Intra-Day ACC (%)	
CAL1	10	8.88	88.84	10.11	101.05	11.15	111.46	100.45
CAL2	15	16.47	109.83	14.54	96.95	14.75	98.36	101.71
CAL3	30	28.95	96.50	30.63	102.12	30.31	101.03	99.88
CAL4	45	44.46	98.80	45.81	101.79	41.94	93.20	97.93
CAL5	60	62.27	103.78	58.63	97.71	61.48	102.46	101.32
CAL6	80	78.63	98.29	80.69	100.86	80.46	100.58	99.91

**Table 2:** Intra-day and inter-day percentages of accuracy (ACC) and coefficient of variation (CV), related to quality controls (QC).

Standard	Nominal Concentration (µg/mL)	First Analytical Session		Second Analytical Session		Third Analytical Session		Inter-Day ACC (%)	Inter-Day CV (%)
		Intra-Day ACC (%)	Intra-Day CV (%)	Intra-Day ACC (%)	Intra-Day CV (%)	Intra-Day ACC (%)	Intra-Day CV (%)		
LQC	12.5	98.59	3.96	101.25	1.41	98.89	4.62	99.58	3.33
MQC	22.5	98.94	7.22	96.17	3.13	94.59	4.27	96.57	4.87
HQC	35	95.79	1.13	92.09	2.72	92.81	4.94	93.56	2.93

**Table 3:** Mean concentration values of the galenic compounds in several storage conditions ± SE and ACC.

	Glass 4 °C		PET 4 °C		Glass RT		PET RT		Glass 45 °C		PET 45 °C	
Time	Mean ± SE (μg/mL)	ACC (%)	Mean ± SE (μg/mL)	ACC (%)	Mean ± SE (μg/mL)	ACC (%)	Mean ± SE (μg/mL)	ACC (%)	Mean ± SE (μg/mL)	ACC (%)	Mean ± SE (μg/mL)	ACC (%)
0	534.44 ± 0.22	106.89	534.44 ± 0.22	106.89	534.44 ± 0.22	106.89	534.44 ± 0.22	106.89	534.44 ± 0.22	106.89	534.44 ± 0.22	106.89
24 h	521.55 ± 15.44	104.31	495.90 ± 8.34	99.18	511.62 ± 15.57	102.32	510.21 ± 11.23	102.04	533.15 ± 2.93	106.63	522.45 ± 17.79	104.49
48 h	510.14 ± 16.21	102.03	499.36 ± 18.46	99.87	532.79 ± 16.32	106.56	472.93 ± 5.47	94.59	489.08 ± 7.34	97.82	474.57 ± 4.49	94.91
72 h	481.27 ± 14.85	96.25	527.04 ± 11.13	105.41	478.11 ± 9.41	95.62	512.36 ± 30.61	102.47	491.57 ± 8.70	98.31	498.20 ± 18.19	99.64
1 w	542.82 ± 5.47	108.56	520.26 ± 18.92	104.05	535.46 ± 3.81	107.09	522.31 ± 3.12	104.46	523.77 ± 5.70	104.75	544.25 ± 1.59	108.85
2 w	482.96 ± 14.52	96.59	495.74 ± 4.13	99.15	499.95 ± 1.44	99.99	515.07 ± 7.09	103.01	499.49 ± 3.30	99.90	502.41 ± 19.46	100.48
1 m	549.93 ± 2.33	109.99	490.14 ± 2.58	98.03	509.59 ± 10.81	101.92	523.13 ± 5.21	104.63	527.81 ± 7.58	105.56	526.58 ± 7.62	105.32
2 m	493.20 ± 5.10	98.64	506.61 ± 4.60	101.32	503.96 ± 4.13	100.79	513.96 ± 1.15	102.79	468.86 ± 5.55	93.77	499.53 ± 4.62	99.91
3 m	498.00 ± 5.36	99.60	499.40 ± 3.94	99.88	502.24 ± 4.28	100.45	496.21 ± 2.11	99.24	442.56 ± 1.29	88.51	481.68 ± 4.79	96.34
4 m	492.21 ± 2.35	98.44	484.68 ± 3.71	96.94	488.47 ± 2.98	97.69	497.41 ± 5.58	99.48	423.64 ± 6.28	84.73	471.23 ± 9.59	94.25
5 m	487.48 ± 2.25	97.50	494.97 ± 5.46	98.99	480.44 ± 7.87	96.09	492.89 ± 2.86	98.58	421.57 ± 3.39	84.31	485.18 ± 11.77	97.04
6 m	503.53 ± 7.84	100.71	492.02 ± 4.12	98.40	469.81 ± 6.35	93.96	515.66 ± 3.51	103.13	420.27 ± 1.97	84.05	499.67 ± 2.98	99.93
7 m	483.09 ± 1.24	96.62	493.05 ± 2.66	98.61	466.2 ± 5.74	93.26	509.0 ± 7.79	101.82	419.4 ± 7.31	83.90	516.9 ± 8.70	103.39

**Table 4:** Compound identified by LC-MS in the degraded compound.

Compound	Molecular Formula	Retention Time (min)	Identified Ion Mass (*m*/*z*)
Glycopyrrolate	C_19_H_28_NO_3_	4.738	[M+H]^+^ 318.2062
Metabolite M9 α-cyclopentylmandelic acid	C_13_H_16_O_3_	10.10	[M+H-H_2_O]^+^ 203.1066

**Table 5:** Median area determined for glycopyrrate and metabolite M9 in the compound stored at 45 °C and 4 °C determined for glycopyrrolate in the compound stored at 45 °C (sample) and 4 °C (control). The ratio between sample and control, calculated for both compounds, is also reported together with the corresponding *p*-value.

Compound	Median Area ± SD (Glass 45 °C = Sample)	Median Area ± SD (Glass 4 °C = Control)	Ratio Sample/Control	*p*-Value
Glycopyrrolate	6.86 × 10^7^ ± 2.75 × 10^6^	8.93 × 10^7^ ± 2.23 × 10^6^	0.768	7.0 × 10^−6^
Metabolite M9	1.50 × 10^6^ ± 1.08 × 10^5^	1.78 × 10^6^ ± 4.81 × 10^4^	1.187	1.1 × 10^−2^

## Data Availability

Data are available upon reasonable request to the corresponding author.
